# Marine and non-marine strata preserving Ediacaran microfossils

**DOI:** 10.1038/s41598-019-46304-7

**Published:** 2019-07-08

**Authors:** Ilana Lehn, Rodrigo Scalise Horodyski, Paulo Sérgio Gomes Paim

**Affiliations:** Universidade do Vale do Rio dos Sinos, Geology Graduate Program - Av. Unisinos, 950, Cristo Rei, Caixa-postal: 275, 93022-000 São Leopoldo, RS Brazil

**Keywords:** Sedimentology, Palaeontology

## Abstract

We report the first occurrence of microfossils in Ediacaran strata of the Camaquã Basin. The assemblage includes simple (*Leiosphaeridia* sp. predominantly) and ornamented acritarchs associated with microbial mats. They are related to the Ediacaran Complex Acanthomorph Palynoflora (ECAP) and Late Ediacaran Leiosphere Palynoflora (LELP) due to the similar morphology and time interval assigned to those assemblages, though the observed specimens are a lot simpler and less diversified. However, different from the usual occurrences, this case study reports Neoproterozoic cosmopolitan communities living in marine (basal unit) and lacustrine (middle units) settings. Fossils within non-marine strata in the Precambrian record are rare. Therefore, this first finding of microfossils in the Camaquã Basin constitutes a new piece of the puzzle related to the history of the Panafrican-Brasiliano basins and shed some light on possible settings where the Ediacaran eukaryotes have evolved.

## Introduction

Precambrian sedimentary basins have been intensively investigated all around the world in terms of eukaryotic protists that arise in the Late Paleoproterozoic Era but intensively diversify during the Ediacaran Period. The Proterozoic life is generally regarded as dominated by marine and intertidal biota preserved in both carbonate^1,2^ and siliciclastic^3,4^ rocks. However, a few studies also indicate that Precambrian land surfaces housed biota in paleokarst^5^, lateritic paleosols^6^, lacustrine^7^ and subaerial non-marine settings^8^. Actually, non-marine, organic-walled microfossils identified as acritarchs were first described long ago, in a Torridonian sequence of northwest Scotland^9^, and were later refined by other authors^10,11^.

Given the particular importance of Neoproterozoic biota for the comprehension of the spreading of life across diverse ecological niches, we register the first occurrence of Ediacaran, organic-walled microfossils preserved in fine-grained siliciclastic strata of the Camaquã Basin, southernmost Brazil. Furthermore, dealing with an Ediacaran succession, most of the forms are simple and low-diversity, but related to both marine and lacustrine settings, hence representing cosmopolitan microorganisms.

## Results

**Figure 1:**
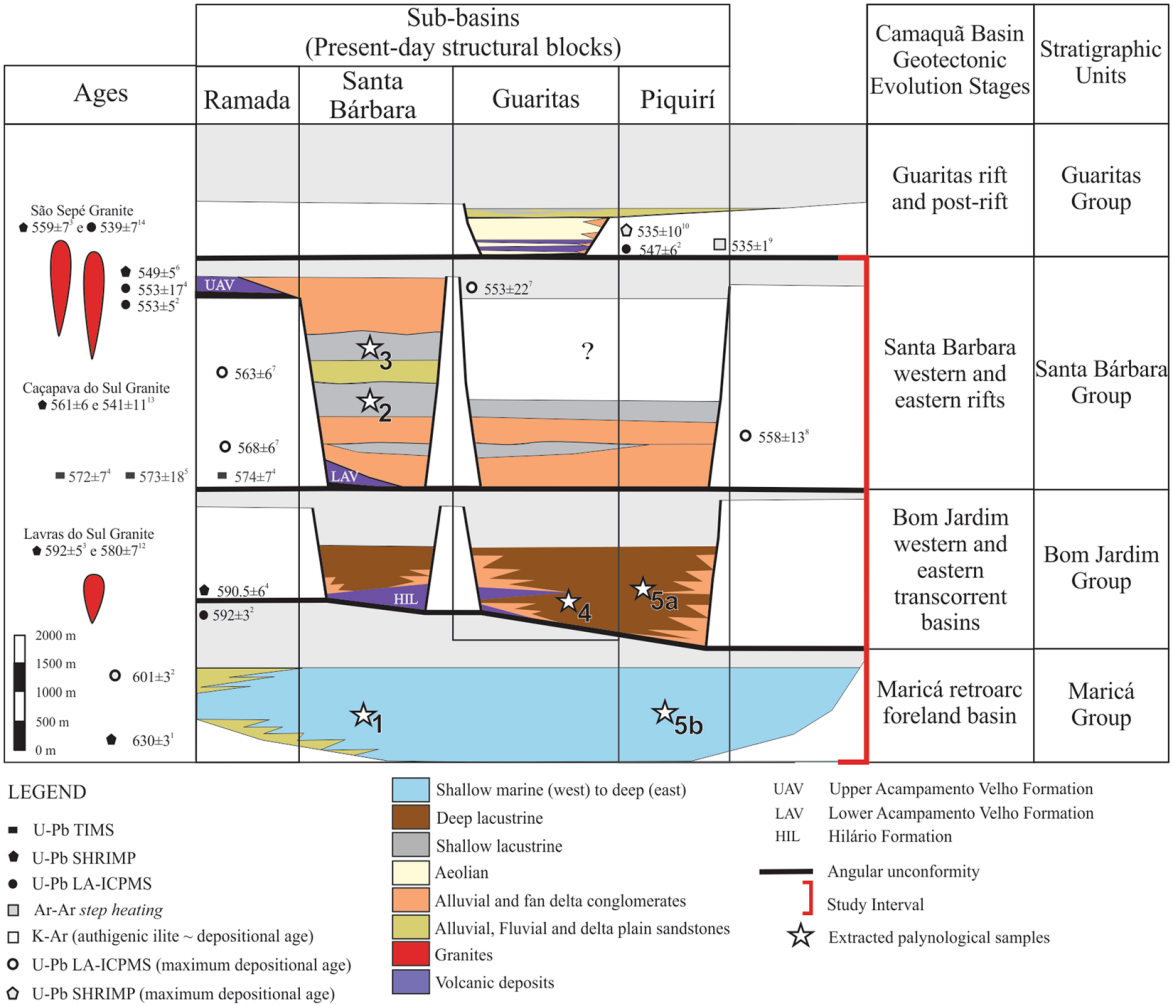
Tectono-sedimentary evolution of the Camaquã Basin (modified from 12). White stars represent stratigraphic distribution of the sampled outcrop sets (see Supplementary File): 1- Outcrop set 1: Maricá Group – Santa Bárbara sub-basin; 2 – Outcrop set 2: Santa Bárbara Group - Santa Bárbara sub-basin, base; Outcrop set 3: Santa Bárbara Group- Santa Bárbara sub-basin, top; Outcrop set 4: Bom Jardim Group – Guaritas sub-basin; Outcrop set 5a: Bom Jardim Group - Piquirí sub-basin; Outcrop set 5b: Maricá Group – Piquirí sub-basin. Radiometric ages are assigned to specific authors in Paim *et al*.^12^.

**Figure 2:**
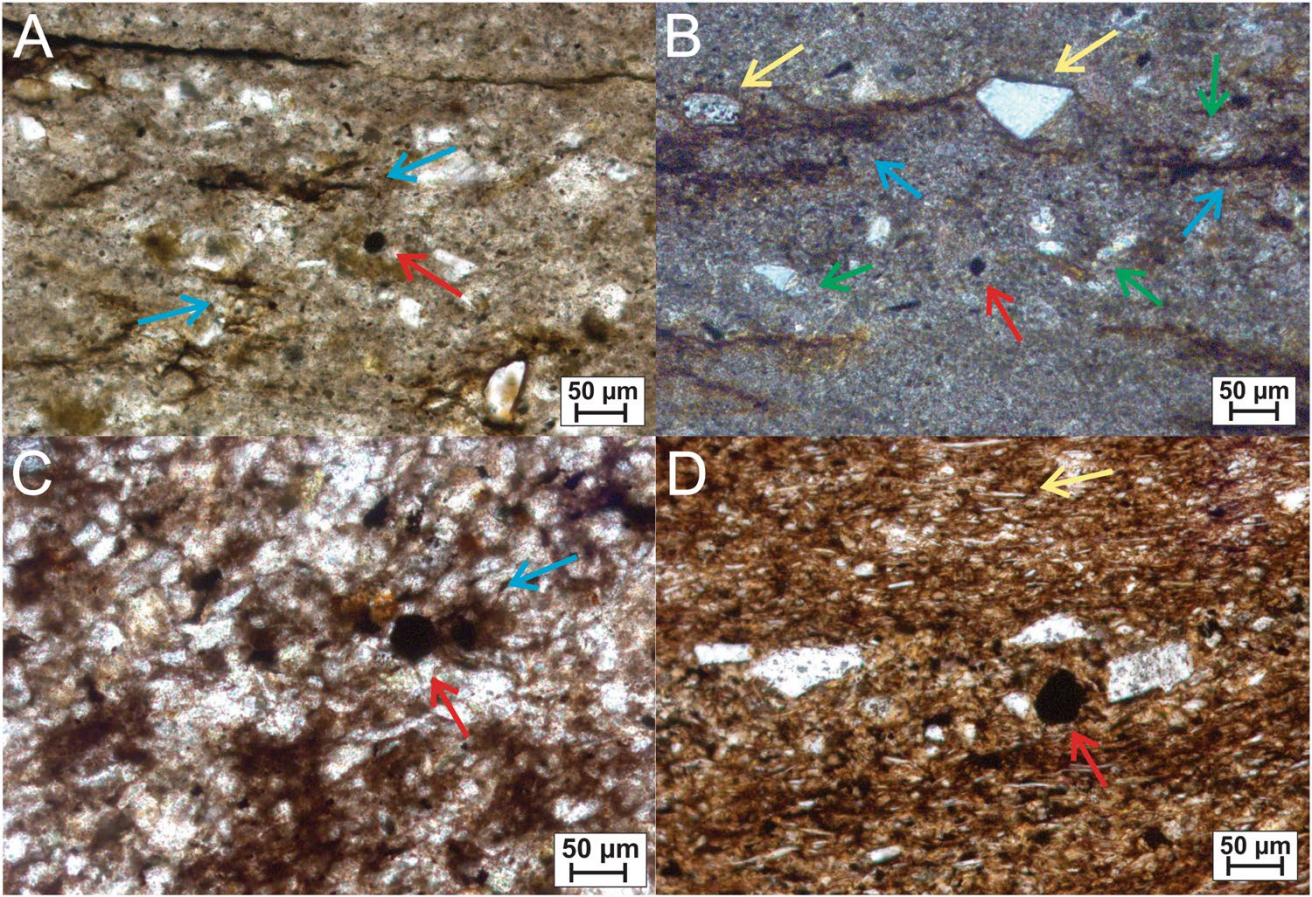
Photomicrograph of sedimentary features and microfossil content. (**A**) Fine-grained facies of Maricá Group seen under natural light showing a sphaeromorph acritarch (red arrow), filamentous bacterial mats (blue arrows), and carbonate cement around the grains; (**B**) Fine-grained facies form Maricá Group seen under polarized light showing a sphaeromorph acritarch (red arrow), grains trapped (yellow arrows) by filamentous bacterial mats (blue arrows), and carbonate cement around the grains (green arrows). (**C**) Simple spherical microfossil (red arrow) and silt grains involved by filamentous bacterial mats (blue arrow) in the fine-grained facies of the Bom Jardim Group; (**D**) Siltstone of the Santa Bárbara Group displaying aligned grains of mica (yellow arrow) and opaque rounded microfossil (red arrow) inside a fossilized microbial mat.

Even though the Camaquã Basin (Fig. 1) ichnological content is studied since the 1990’s^12–15^, this is the first record of body fossils on it. Microfossils occur in siliciclastic mudstones of the Maricá, Bom Jardim and Santa Bárbara groups (Fig. 2). This fossil record comprises abundant acritarchs, identified as simple and ornamented forms and remains of bacterial mats.

Siltstones and very fine-grained sandstones of the Maricá Group are immature and represent storm deposits. Grains are angular to subangular and eventually entrapped by filamentous microbial mats (Fig. 2A,B). Different from the other units, they do not present eodiagenetic hematite coating, but only poikilotopic calcite cement. Although also immature, siltstones and very fine-grained sandstones of both Bom Jardim and Santa Bárbara groups record hyperpycnal turbidity currents. Grains are also angular to subangular, but contain eodiagenetic hematite coatings precipitated before carbonate cementation has taken place (Fig. 2C,D).

### Systematic palaeontology

#### Repository

Described palynological slides are kept in the Museu de História Geológica do Rio Grande do Sul - MHGEO – Unisinos, São Leopoldo, RS, Brazil – and assigned numbers according to the collection (ULVG – UNISINOS/Laboratório de História da Vida e da Terra - Lavigea). Location and geographic coordinates for each sample are presented in the Supplementary File.

Incertae sedis

Group **Acritarch** Evitt, 1963

Genus ***Leiosphaeridia****,* Eisenack, 1958, emend. Turner, 1984,

Type species ***Leiosphaeridia baltica****,* Eisenack, 1958

*Leiosphaeridia* spp.

**Figure 3:**
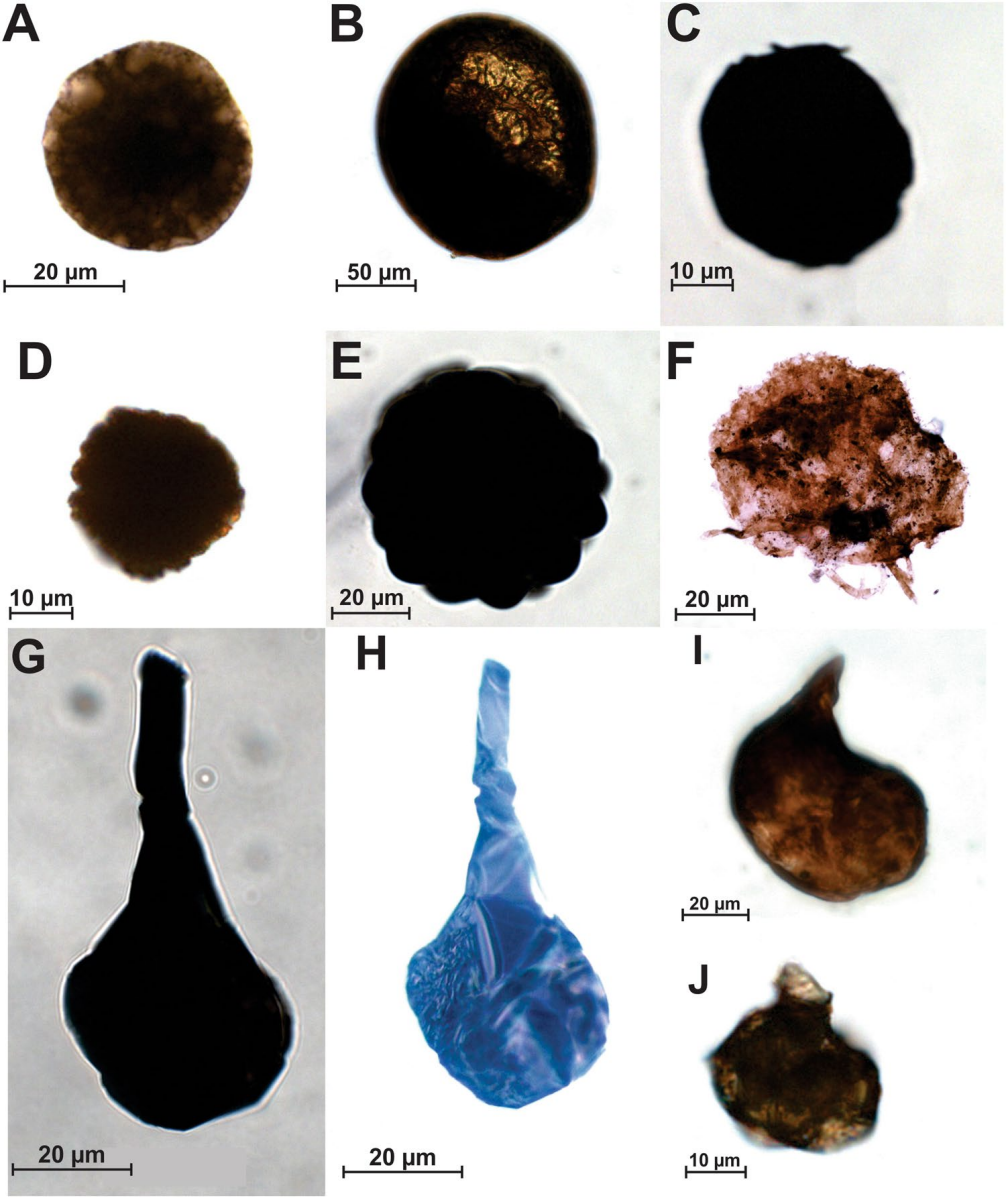
Acritarchs view under transmitted light microscopy. (**A**–**C**) *Leiosphaeridia* sp. from (**A**) Maricá (sample ULVG 12518), (**B**) Bom Jardim (sample ULVG 12495), and (**C**) Santa Bárbara (sample ULVG 12511) groups; (**D**,**E**) *Lophosphaeridium sp*. from (**D**) Maricá (sample ULVG 12518) and (**E**) Bom Jardim (sample 12495) groups; (**F**), *Tanarium irregulare* from Santa Bárbara Group (sample 12487); (**G**–**J**), *Germinosphaera sp*. from (**G**,**H**) Maricá (sample ULVG 12509), original opaque (**G**) and illuminated (**H**) image, (**I**) Bom Jardim (sample ULVG 12506), and (**J**) Santa Bárbara (sample ULVG 12515) groups.

**Figure 4:**
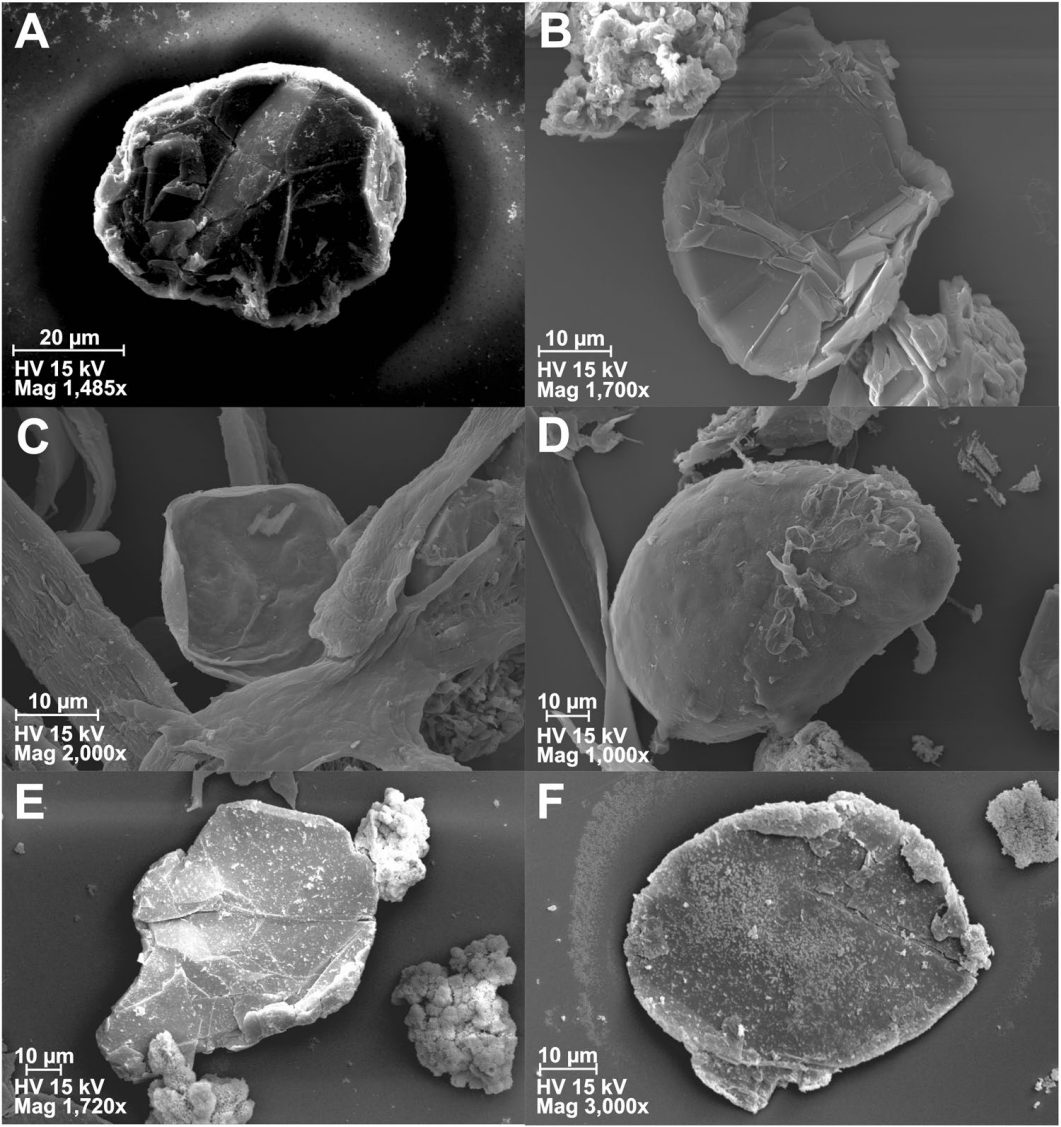
Acritarchs view on scanning electron microscopy. (**A**,**B**) *Leiosphaeridia* sp. from Maricá Group (sample ULVG 12509); (**C**,**D**) *Leiosphaeridia sp*. (**C**) and *Lophosphaeridium sp*. (**D**) with filamentous bacteria from Bom Jardim Group (sample ULVG 12506); (**E**,**F**) *Germinosphaera sp*. (**E**) and *Leiosphaeridia sp*. (**F**) from Santa Bárbara Group (sample ULVG 12508) with bacterial mats fragments. Note folded microfossils due to compaction (**B**,**C** images).

Figures 3A–C and 4A–C/F

#### Occurrence and material

Slides ULVG 12495, 12496, 12505, 12506, 12507, 12508, 12511, 12513, 12514, 12517, 12518, 12519, 12520. One hundred forty nine complete specimens and dozens of fragments of sphaeromorphs were extracted from mudstones of the Maricá, Bom Jardim and Santa Bárbara outcrops (see Supplementary File - Table ST1).

#### Description

Organic-walled, acid-resistant, simple spherical to sub-spherical, compressed, commonly folded vesicles with smooth to shagrinate or granular wall surface. Diameter ranging from 20 to 150 µm.

#### Remarks

Some species show excystment opening, median split or partial rupture^16^. *Leiosphaeridia* is a long-ranging genus, ranging from Paleoproterozoic until present. This genus is the most abundant microfossil in the geologic record, with a great variability of vesicle dimensions^17,18^. The group comprises four basic species based on the wall thickness and size class: *L. minutissima* – thin-walled, <70 µm; *L. tenuissima* – thin-walled, 70–200 µm; *L. crassa* – thicker-walled, <70 µm; *L. jacutica* – thicker-walled, 70–800 µm^19^.

#### Discussion

The Camaquã Basin palynomorphs includes more than one leiosphaerid species, but it is impossible to assert which species because the poor preservational quality precludes the identification of wall internal details and thickness. Based on the size of the recovered specimens it is possible to associate then with two potential species: *L. minutissima* and *L. crassa*. However, the lack of diagnostic features about wall-thickness within this group of acritarchs makes difficult to ascribe them to species level.

Genus ***Tanarium*** Kolosova, 1991,

emend. Moczydlowska, Vidal and Rudavskaya, 1993

Type species ***Tanarium conoideum*** Kolosova, 1991,

emend. Moczydlowska, Vidal and Rudavskaya, 1993

#### Description

Organic-walled, acid-resistant microfossils consisting of spherical to sub-spherical vesicle bearing numerous hollow, cylindrical processes distributed around the vesicle outline. The processes are more or less of equal size (in a single specimen), differing in number between specimens and not very evenly distributed. Diameter ranging from 80 to 200 µm.

***Tanarium irregulare*** Moczydlowska, Vidal and Rudavskaya, 1993

Figure 3F

#### Occurrence and material

Slides ULVG 12487, 12493, 12514, 12515. Six complete and dozens of fragmented vesicles appear in palynological material extracted from mudstones of the Maricá, Bom Jardim and Santa Bárbara outcrops (see Supplementary File - Table ST1).

#### Description

Organic-walled, acid-resistant microfossils consisting of originally spherical to sub-spherical vesicles irregular in outline ornamented with long, tubular and conical, simple and branching protrusions. Diameter ranging from 50 to 150 µm and spines ranging from 10 to 20 µm.

#### Remarks

The specimens are associated with diagnosis characteristics of *Tanarium irregular* sp.^20,21^. The name derives from Latin *irregularis* – irregular, referring to the processes shape.

Genus ***Lophosphaeridium*** Timofeev, 1959,

emend. Downie, 1963, emend. Lister, 1970

Type species ***Lophosphaeridium rarum*** Timofeev, 1959,

emend. Downie, 1963

*Lophosphaeridium* sp.

Figure 3 D-E and 4D

#### Occurrence and material

Slides ULVG 12494, 12506, 12507, 12509, 12510, 12511, 12513, 12515. Twenty-six entire and dozens of fragmented specimens extracted from mudstones of the Maricá, Bom Jardim and Santa Bárbara outcrops (see Supplementary File - Table ST1).

#### Description

Organic-walled, acid-resistant vesicle, circular to sub-circular in outline. Compactional folds present on some specimens. The surface of the vesicle displays numerous tightly arranged, small conical spines that are visible as evenly small hairs distributed around the vesicle outline. Vesicle diameter ranging from 50–150 µm. Conical projections are approximately 10–15 µm long.

#### Remarks

The studied specimens are comparable to *Lophosphaeridium* sp.^22–24^ regarding their morphological features and size, but it not possible to ascribe them to species level.

Genus ***Germinosphaera*** Mikhailova, 1986, emend. Butterfield, 1994

Type species ***Germinosphaera bispinosa*** Mikhailova, 1986

*Germinosphaera* sp.

Figures 3G–J and 4E

#### Occurrence and material

Slides ULVG 12487, 12498, 12503, 12505, 12494, 12506, 12507, 12509, 12510, 12511, 12513, 12515, 12517, 12518, 12519, 12520. Forty-two microfossils extracted from mudstones of Maricá, Bom Jardim and Santa Bárbara outcrops (see Supplementary File - Table ST1).

#### Description

Organic-walled, acid-resistant spheroidal vesicles with one or more open-ended, tubular and occasionally branched processes that communicate freely with the vesicle. Bodies 70–100 µm long and 30–70 µm wide, occasionally with a vase-shaped form with short or elongated processes, 5–15 µm wide and 10–50 µm.

#### Remarks

All complete specimens of *Germinosphaera* sp.^25,26^ found in the analysed samples bear a single filamentous process.

## Discussion

Fine-grained facies of the Camaquã Basin show organic-walled microfossils identified as simple and ornamented acritarchs as well as bacterial mats (filamentous and coccoidal). These organisms occur along the three basal units of the Camaquã Basin: Maricá, Bom Jardim and Santa Bárbara groups. Each unit record a distinct tectonic setting, which led to different environmental conditions^27^ (Fig. 1). The fine-grained strata of the Maricá Group record a short-distance transport of immature, angular to sub-angular sediments deposited under wave action in a shallow marine setting (See Supplementary File). No micro (hematite coating) or macroscopic (desiccation cracks) features related to subaerial exposure were identified. Similarly, both Bom Jardim and Santa Bárbara groups also comprise immature, short-distance transported sediments deposited in a subaqueous realm. However, a much larger alluvial influence (relative to the Maricá Group), and associated hyperpycnal turbidity currents, and rare (Bom Jardim Group) to common (Santa Bárbara Group) subaerial exposure of the depositional surface suggest continental, deep- to shallow lacustrine settings (See Photographs in Supplementary File). Accordingly, eodiagenetic hematite coatings are widespread in both units.

Camaquã Basin microfossils can be associated with Ediacaran Complex Acanthomorph Palynoflora (ECAP)^28^ in aspects of time range and with Late Ediacaran Leiosphere Palynoflora (LELP)^29^ when observed morphological aspects. This association still needs an improvement because nor the time range distribution or the morphology of the organisms are equal of the described assemblages. Camaquã Basin microfossils seems to represent a new group of microfossils (Fig. 3).

The same assemblage was recovered in both marine (Maricá Group) and lacustrine settings (Bom Jardim and Santa Bárbara groups). Although acritarchs have usually been associated with marine settings, this taxonomic overlap is frequent among microfossil taxa from marine and non-marine setting in the Precambrian^9,10,30^ and reinforces the idea that morphological simple genera such as *Leiosphaeridia* not necessarily indicate any particular environment^11^. Some species of *Leiosphaeridia* may be related to the Prasinophyceae^31,32^, but the simple morphology of this genus does not preclude a wide variety of natural affinities and sources, both marine and non-marine^30^. Besides, organic-walled sphaeromorphs were also found in non-marine Torridonian rocks of Scotland^9,11^. Other works described similar organisms in non-marine strata and proposed a Proterozoic terrestrial colonization^7,8,33^ as well as a continental evolution for the Eukarya during the Late Ediacaran^11^.

The recovered microfossils were always associated with microbial mat fragments, as demonstrated in palynological slides (Fig. 4) and thin sections (Fig. 2). This coincidence reinforces previous suggestions that many Ediacaran acritarchs could represent benthic and even heterotrophic life stages^30–32^. We understand that this close association is not a definitive proof. There is the possibility of planktonic organisms sinking on the bottom and being trapped by bacterial mats. However, why microfossils do not occur when microbial mats are absent? Therefore, this close association does suggest a co-existence of both microfossils and microbial mats. Searching for favourable conditions and food, in a stable sea or lacustrine floor, with no signs of transport, these microorganisms inhabit the same subaqueous environment where microbial colonies build up mats.

We demonstrate that the first find of body fossils in the Camaquã Basin add new information about the Ediacaran fossil record of South America and fill some gaps about microfossils existence and distribution in a Proto-Gondwana context. Several Ediacaran basins as Nama (Namibia), Arroyo del Soldado (Uruguay), Corumbá (Brazil) and others register body fossils related to ECAP and ELP assemblages. Even simpler and less diversified that those assemblages, the Camaquã Basin microfossils reported here include Camaquã Basin as hostess of Ediacaran life at southwestern Proto-Gondwana.

The recovery of microfossils in marine and lacustrine strata and the profusion of Leiosphaerids suggest that these polyphyletic long-ranging taxa are cosmopolitan, as suggested by previous works^11,33,34^. Besides, the lack of macrofossils of the Ediacara biota suggests restraining ecological conditions (large fluvial inflow) for both marine and lacustrine settings developed during basin evolution. In addition, the Ediacara macrofossils occur in Late Ediacaran strata (575–542 Ma) around the world^1^. This time interval is compatible with the Santa Bárbara Group, which reflect shallow-lacustrine conditions, favourable for cosmopolitan microrganisms.

## Conclusions

The first finding of body fossils in the Camaquã Basin adds new evidence about the Ediacaran fossil record of South America, improves the dataset and complements the scenario of life within the Proto-Gondwana. Based on their similar morphology and assigned time interval the reported microfossils could be ascribed to the Ediacaran Complex Acanthomorph Palynoflora (ECAP) and Late Ediacaran Leiosphere Palynoflora (LELP), even though the observed specimens are simpler and less diversified.

The finding of microfossils in marine and lacustrine strata and the profusion of Leiosphaerids suggest that both habitats were already colonized by cosmopolitan eukaryotes by the Late Ediacaran. On the other hand, the lack of typical components of the Ediacara fauna suggest restrictive ecological conditions in both marine and lacustrine realms during the Camaquã Basin evolution. At last, the close relationship between microfossils and bacterial mats suggest a possible link between both components of the Camaquã Basin biota and their living conditions.

## Material and Methods

This study includes forty-three palynological slides obtained from outcrop samples. The outcrops record distinct intervals sampled at different locations (see Supplementary File - ST1). All samples are stored in the collection of the Museu de História Geológica do Rio Grande do Sul of the Universidade do Vale do Rio dos Sinos (São Leopoldo, Brazil). The palynological preparation technique was used for the extraction of acid-insoluble microfossils. Following Grey’s technique^35^, raw samples were mechanically disaggregated. After, they were digested with HCl and HF for carbonate and silicate removal, respectively. Boiling HCl was used for the removal of clay minerals. Strewed kerogen was oxidized with concentrated HNO3. After filtration (10 µm filter size) and swirling to separate heavy minerals, strew slides were prepared and examined under transmitted light microscope with interference contrast (Zeiss Axio Imager-A2). SEM-EDS (scanning electron microscopy plus energy-dispersive X-ray spectroscopy) analyses were performed on gold-coated samples obtained from representative microfossil specimens. SEM studies were executed at Instituto Tecnológico de Micropaleontologia - itt FOSSIL - of the Universidade do Vale do Rio dos Sinos – UNISINOS (São Leopoldo City/Rio Grande do Sul State), using a Zeiss EVO/MA15 SEM equipment, at Laboratório de Conformação Nanométrica – Instituto de Física of the Universidade Federal do Rio Grande do Sul – UFRGS (Porto Alegre/Rio Grande do Sul), using a JIP-4500 MultiBeam SEM-FIB equipment and at Laboratório Nacional de Nanotecnologia – LNNano - of the Centro Nacional de Pesquisa em Energia e Materiais – CNPEM (Campinas/São Paulo), using a Quanta 650FEG SEM equipment.

## Supplementary information

Supplementary File

## Data Availability

All data generated or analysed during this study are included in this published article and in the Supplementary File.
