# Supplementary material for: Marine and non-marine strata preserving Ediacaran microfossils
Source: Sci Rep. 2019 Jul 8;9:9809. doi: 10.1038/s41598-019-46304-7 (PMC6614404; doi:10.1038/s41598-019-46304-7)
Supplement: Supplementary file 1 — Supplementary File [file 41598_2019_46304_MOESM1_ESM.docx]

**Marine and non-marine strata preserving Ediacaran microfossils**

Ilana Lehn^a*^, Rodrigo Scalise Horodyski^a^, Paulo Sérgio Gomes Paim^a^

*^a^ Universidade do Vale do Rio dos Sinos, Geology Graduate Program - Av. Unisinos, 950, Cristo Rei, 93022-000 - São Leopoldo, RS - Brasil - Caixa-postal: 275*

**Supplementary information:**

Geological Setting

The Camaquã Basin comprises a thick sedimentary, Neoproterozoic to Early Paleozoic succession including three volcanic intervals. It records late to post-collisional stages of the Brasiliano Orogeny (1, 2, 3).


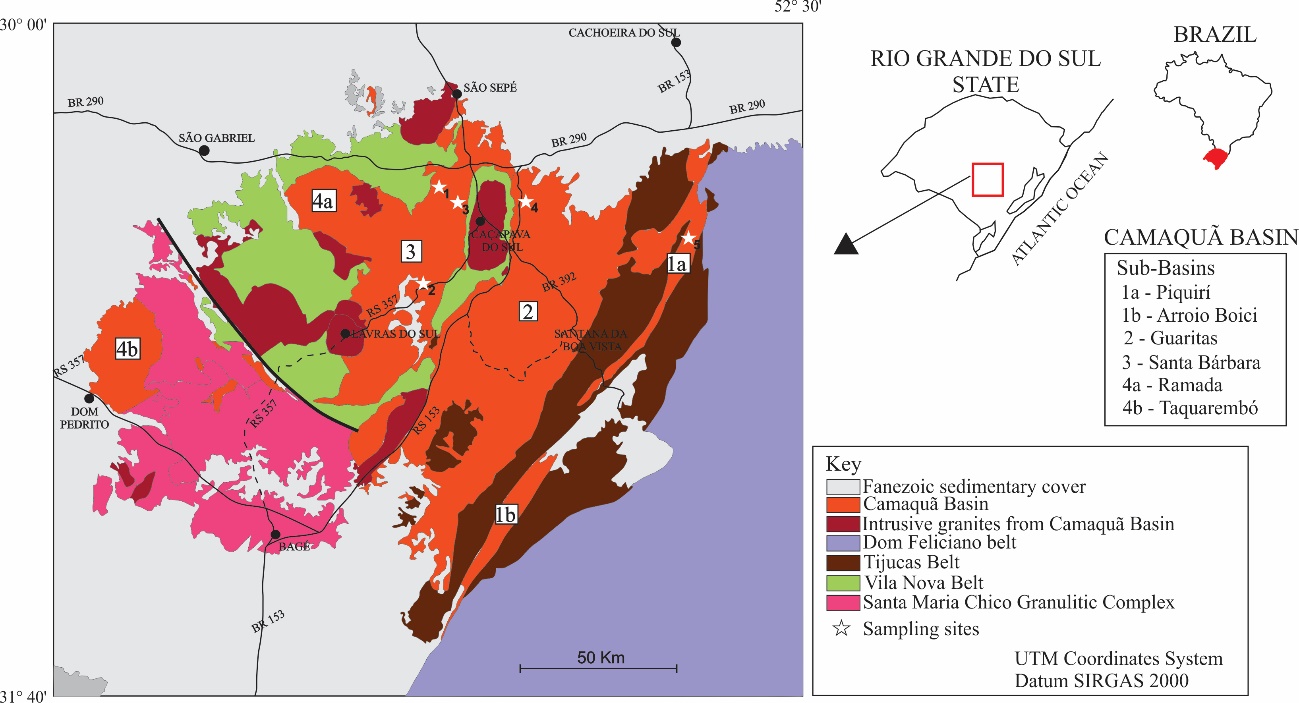


Figure 1. Geological map and geographic distribution of investigated outcrops in the Camaquã Basin, Brazil (modified from 4). See supplementary file for specific sampling sites location.

The geological record crops out along Piquirí–Arroio Boici, Santa Bárbara, Guaritas and Ramada-Taquarembó (Figure 1) structural blocks or sub-basins (4). They include a partial record of four successive tectonic-volcanic-sedimentary episodes evolving from compressive through transpressive and transtensive to distensive tectonic regimes (5). This tectonic evolution is represented by the Maricá Retroarc Foreland Basin, East and West Bom Jardim Strike-slip basins, East and West Santa Bárbara rifts and Guaritas Rift.

The Maricá Group (Figure 1 in the main text) is an unconformity-bounded unit composed of about 1,700 m of siliciclastic sedimentary rocks (4, 6). Its lower interval comprises braided river sandy deposits. Its intermediate portion encompasses a fine-grained, transgressive-regressive marine succession influenced by storm processes that was sampled for palynological analysis. The upper interval encompasses fluvio-deltaic conglomerates and sandstones (7, 5). Based on detrital zircon age determination the maximum depositional age of this group is 601 ±13 Ma (8). Based on geochronological data from the overlying volcanic rocks (Hilário Formation) the Maricá Group has a minimum depositional age of 592 Ma (1). This group has been ascribed to a retroarc foreland basin (9, 5).

The Bom Jardim Group (Figure 1 in the main text) represents a second unconformity-bounded unit about 4,000 m thick. It consists of a volcano-sedimentary package that includes intermediate to basic volcanic rocks of shoshonitic to high-K, calc-alcaline affiliation as well as volcanoclastic strata (Hilário Formation). These volcanic rocks dominate in southwestern portion of the Camaquã Basin, but thin out and are interbedded with siliciclastic rocks in other areas. The siliciclastic deposits were associated with fan deltas developed along the faulted margins of the lacustrine basins (9, 5). The fine-grained lacustrine facies were sampled for microfossils studies. The U-Pb SHRIMP zircon ages of the volcanic rocks range from 592 (1) to 590.5 ± 5.7 Ma (10). The Bom Jardim Group records the infill of two late- to post-collisional basins (East and West Bom Jardim transcurrent basins) developed within a back-arc context (5).

The Santa Bárbara Group (Figure 1 in the main text) is the third unconformity-bounded unit of the Camaquã Basin. At its base, it contains alkaline, mostly acidic volcanic rocks (Lower Acampamento Velho Formation) that delineate along a large part of the Camaquã Basin the contact between the Bom Jardim and Santa Bárbara groups. The siliciclastic package that overlies the Lower Acampamento Velho Formation comprises intervals composed of conglomerates and sandstones of alluvial to fluvial origin intercalated with packages of fine-grained, deltaic sandstones associated with thin-bedded turbidites and debrites deposited in shallow, often desiccated lacustrine environments (4, 11). These lacustrine facies have also been ascribed to either shallow marine to transitional, tidal-influenced (12) or flood-plain (13) depositional settings. Fine-grained facies were sampled at different levels of the Santa Bárbara Group for palynological analyses. The age of the Lower Acampamento Velho Formation yielded the following maximum depositional ages for the base of Santa Bárbara Group: 573 ±18 Ma (1), 574 ±7 Ma (10). The minimum depositional age of this group comes from the dating of intermediate volcanic rocks (Rodeio Velho Formation) related to the youngest unconformity-bounded unit (Guaritas Group). It ranges from 547 ±6.3 Ma (U-Pb method; 8) to 535 Ma (U-Pb of detrital zircon; 2). The Santa Bárbara Group records the filling of two basins (Santa Bárbara East and West rifts; 5) developed within a transtensional setting during post-collisional stages (4, 14).

Supplementary Table:

Table ST1. It shows outcrop sets location and UTM coordinates of sampling sites.

| **Sample** | **Geologic unit** | **Outcrop set** | **UTM coordinates** (WGS 84 datum) |
| --- | --- | --- | --- |
| ULVG 12487 | Santa Bárbara Group | 3 | 247750 E/6612486 S |
| ULVG 12488 | Santa Bárbara Group |  | 255651 E/6625738 S |
| ULVG 12489 | Santa Bárbara Group |  | 251475 E/6615766 S |
| ULVG 12490 | Santa Bárbara Group |  | 252048 E/6619375 S |
| ULVG 12491 | Santa Bárbara Group |  | 242556 E/6604770 S |
| ULVG 12492 | Santa Bárbara Group |  | 242907 E/6579322 S |
| ULVG 12493 | Santa Bárbara Group |  | 253952 E/6618016 S |
| ULVG 12494 | Santa Bárbara Group |  | 256152 E/6622996 S |
| ULVG 12495 | Bom Jardim Group | 5b | 322827 E/6617008 S |
| ULVG 12496 | Bom Jardim Group | 4 | 272809 E/6624478 S |
| ULVG 12497 | Bom Jardim Group |  | 272809 E/6624478 S |
| ULVG 12498 | Bom Jardim Group |  | 272809 E/6624478 S |
| ULVG 12499 | Bom Jardim Group |  | 272809 E/6624478 S |
| ULVG 12500 | Bom Jardim Group |  | 272724 E/6625842 S |
| ULVG 12501 | Bom Jardim Group |  | 272724 E/6625842 S |
| ULVG 12502 | Maricá Group | 1 | 246464 E/6626191 S |
| ULVG 12503 | Bom Jardim Group | 4 | 272887 E/6624333 S |
| ULVG 12504 | Bom Jardim Group |  | 272772 E/6624975 S |
| ULVG 12505 | Bom Jardim Group |  | 272749 E/6625845 S |
| ULVG 12506 | Bom Jardim Group |  | 272724 E/6625842 S |
| ULVG 12507 | Santa Bárbara Group | 3 | 250873 E/6622319 S |
| ULVG 12508 | Santa Bárbara Group |  | 242482 E/6598048 S |
| ULVG 12509 | Maricá Group | 1 | 247073 S/6626575 S |
| ULVG 12510 | Maricá Group |  | 247172 E/6626610 S |
| ULVG 12511 | Santa Bárbara Group | 2 | 242482 E/6598048 S |
| ULVG 12512 | Santa Bárbara Group |  | 251008 E/6622619 S |
| ULVG 12513 | Maricá Group | 1 | 247172 E/6626610 S |
| ULVG 12514 | Santa Bárbara Group | 2 | 251008 E/6622619 S |
| ULVG 12515 | Santa Bárbara Group |  | 250873 E/6622319 S |
| ULVG 12516 | Santa Bárbara Group |  | 250899 E/6622427 S |
| ULVG 12517 | Maricá Group | 1 | 247073 E/6626575 S |
| ULVG 12518 | Maricá Group |  | 246657 E/6626364 S |
| ULVG 12519 | Maricá Group |  | 246506 E/6626223 S |
| ULVG 12520 | Maricá Group |  | 246657 E/6626364 S |
| ULVG 12521 | Bom Jardim Group | 5a | 323199 E/6617492 S |
| ULVG 12522 | Santa Bárbara Group | 2 | 245550 E/6601947 S |
| ULVG 12523 | Bom Jardim Group | 5a | 319487 E/6609319 S |
| ULVG 12524 | Bom Jardim Group | 5b | 323180 E/6611648 S |
| ULVG 12525 | Bom Jardim Group | 5a | 322323 E/6617702 S |
| ULVG 12526 | Santa Bárbara Group | 2 | 242482 E/6598048 S |
| ULVG 12527 | Bom Jardim Group | 5a | 322943 E/6618147 S |
| ULVG 12528 | Bom Jardim Group | 5b | 323844 E/6617252 S |
| ULVG 12529 | Bom Jardim Group |  | 320592 E/6609578 S |
| ULVG 12530 | Santa Bárbara Group | 2 | 244844 E/6602118 S |

Supplementary Photographs:


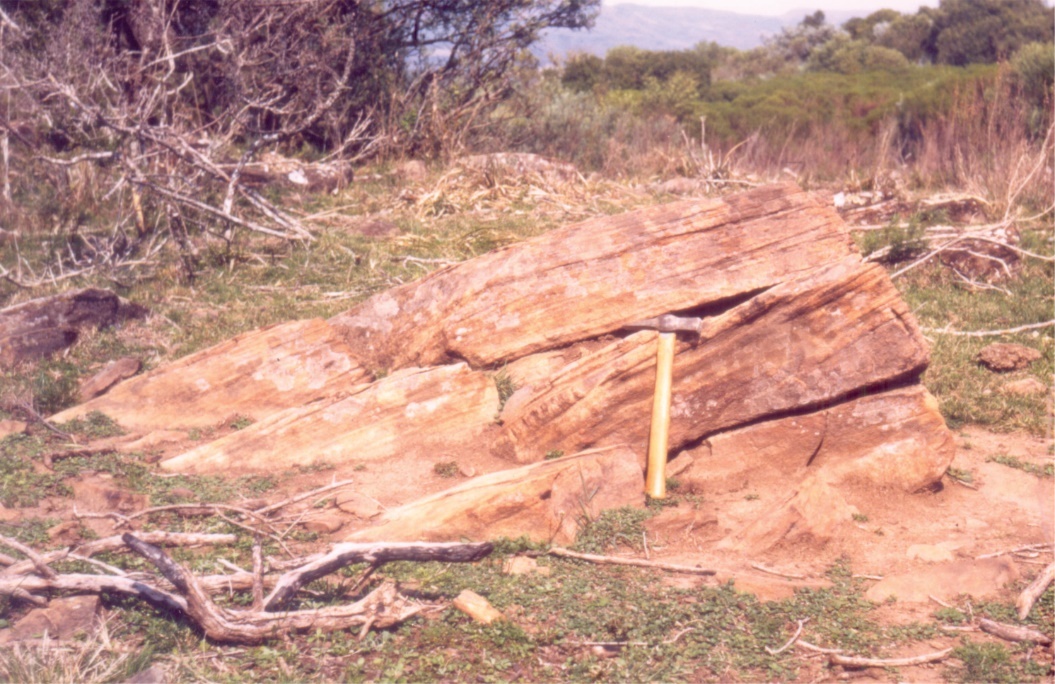


Figure 3 – Well-sorted, fine-grained sandstone with hummocky cross stratification from the Maricá Group (Outcrop set 1).


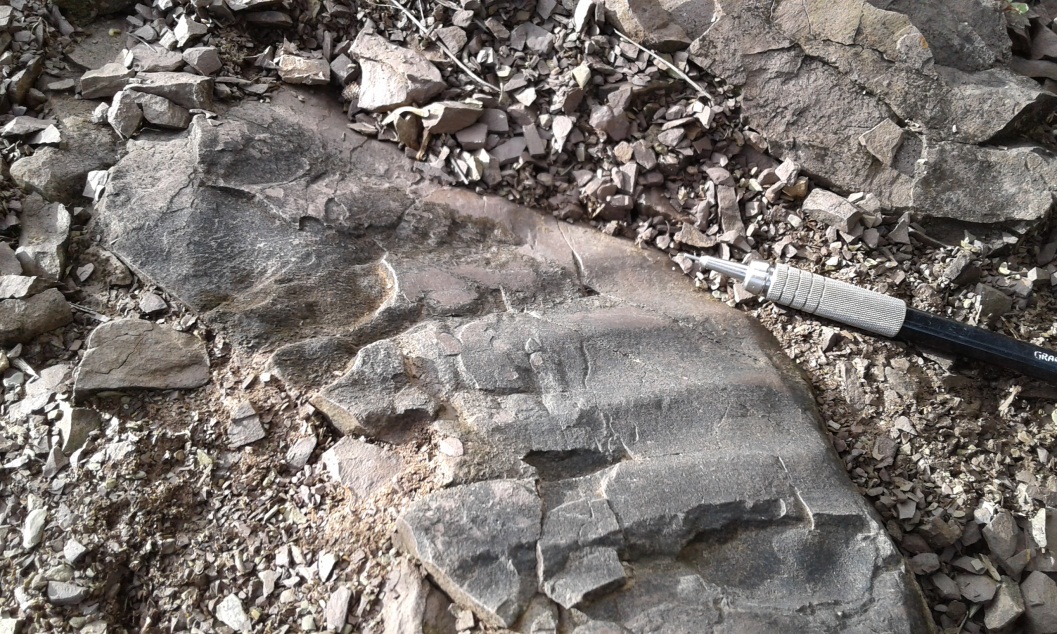


Figure 4- Leveling ripple structures from the Bom Jardim Group (Outcrop set1).


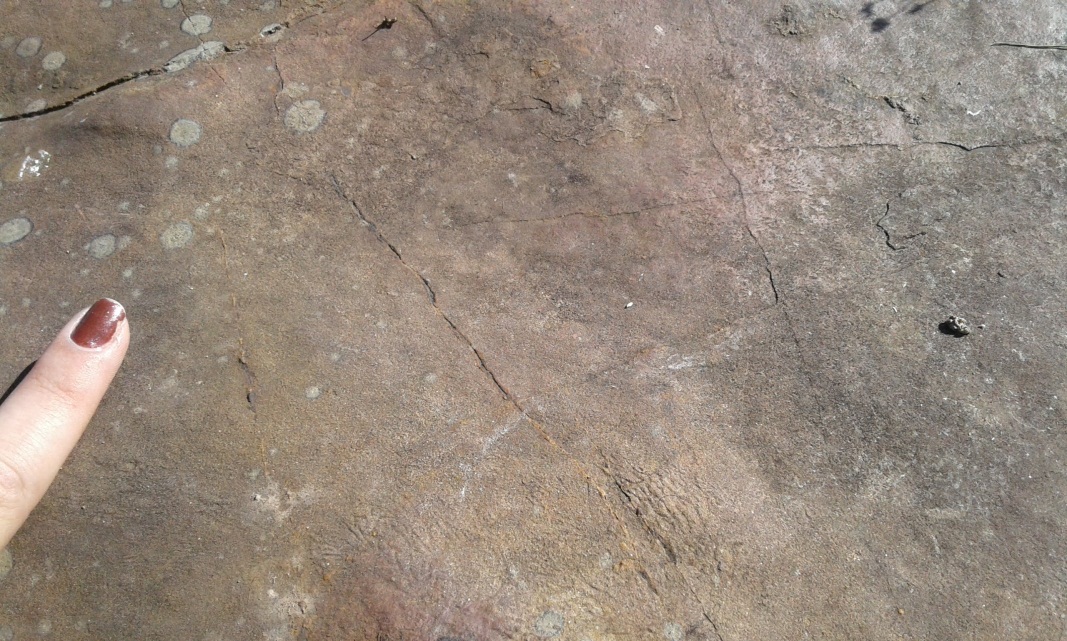


Figure 5 - Remnant/pocket structures from fine-grained deposits of the Bom Jardim Group (Outcrop set 5b).


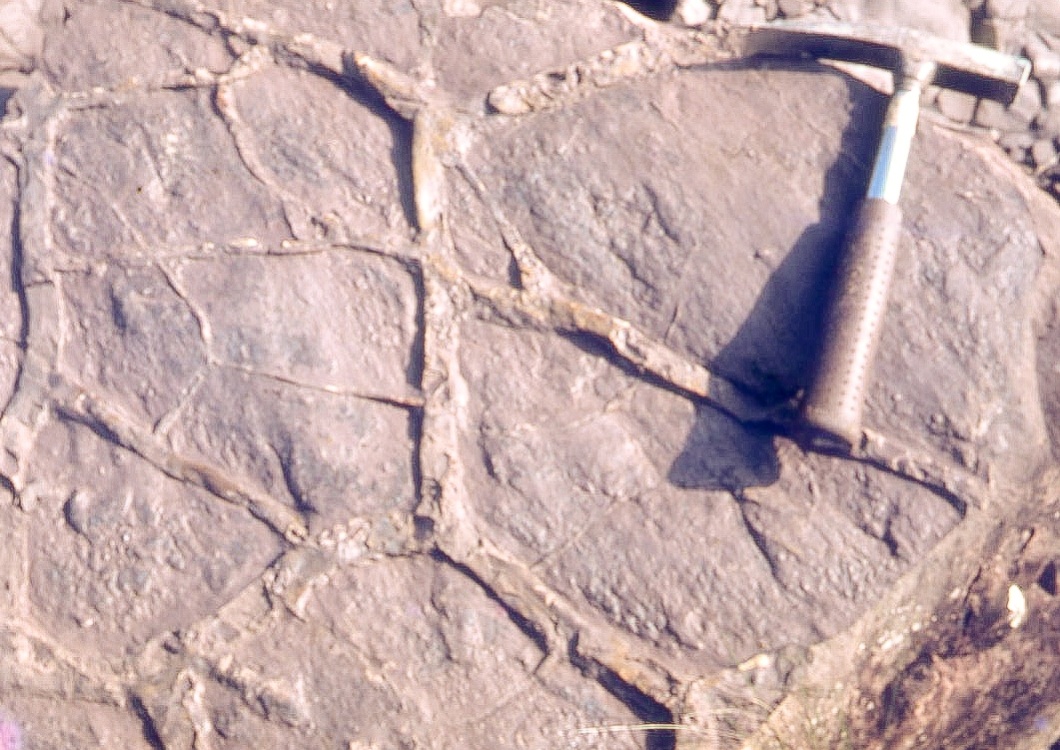


Figure 6 – Desiccation cracks in hyperpycnal turbidites of the Bom Jardim Group (plan view - Outcrop set 4).


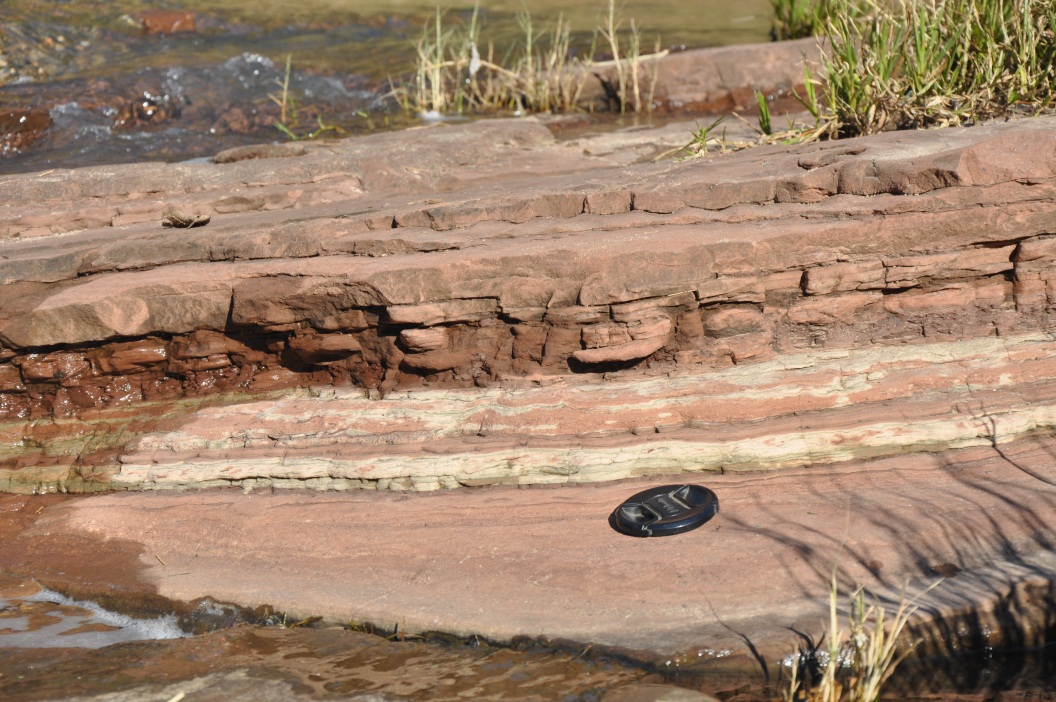


Figure 7 – Fine-grained delta front facies from the Santa Bárbara Group (Outcrop set 3).


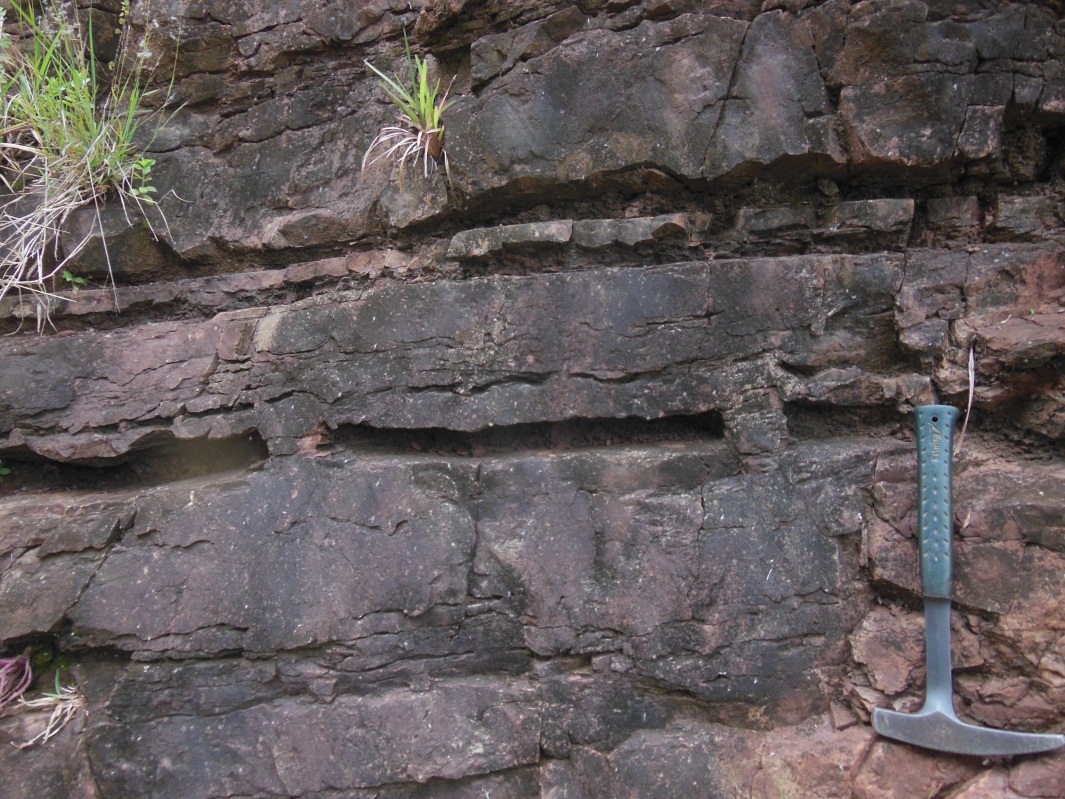


Figure 8 – Desiccation cracks and mud curls in hyperpycnal turbidites of the Santa Bárbara Group (transversal view - Outcrop set 2).


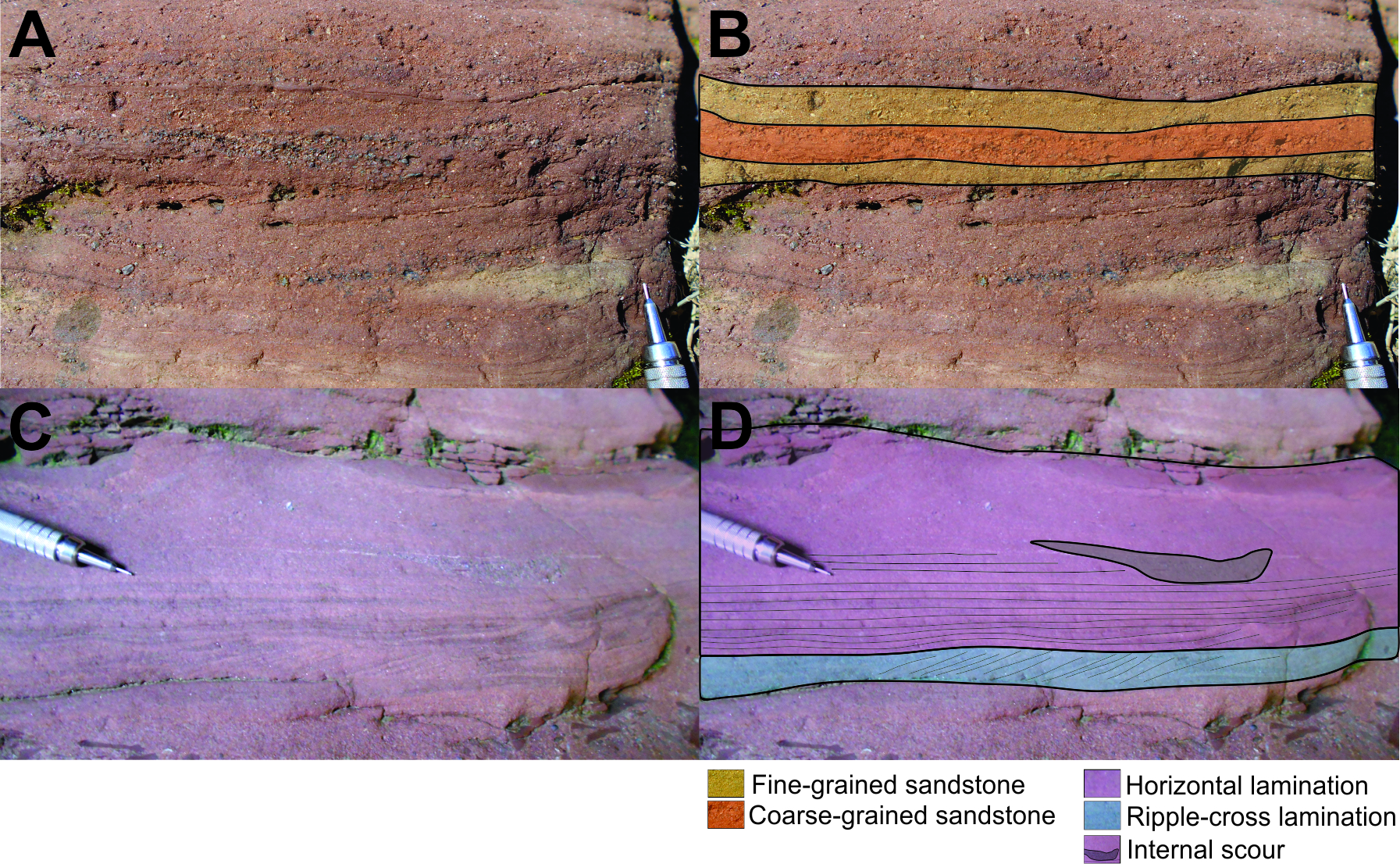


Figure 9 – Original (A and C) and interpreted (B and D) images of hyperpycnal turbidite beds of the Santa Bárbara Group (transversal view - Outcrop set 3).

Supplementary references

1. Almeida, F. Diferenciação Tectônica da Plataforma Brasileira. In: Congr. Bras. Geol., 1, Salvador, 29–46 (1969).
2. Chemale Jr., F. Evolução geológica do Escudo Sul-rio-grandense. In *Geologia do Rio Grande do Sul, (*Holz, M. and De Ros, L. F. Eds.) 13-52, Universidade Federal do Rio Grande do Sul, Porto Alegre, Brazil (2000).
3. Hartmann, L. A., Santos, J. O. S., McNaughton, N. J. Detrital zircon U-Pb age data, and precambrian provenance of the paleozoic Guaritas formation, southern Brazilian shield. *Int.* *Geol. Rev*. 50, 364–374 (2008).
4. Paim, P.S.G., Chemale Jr., F., Lopes, R.C. A Bacia Do Camaquã. In Geologia Do Rio Grande Do Sul. (Holz, M. and De Ros, L. F. Eds.), CIGO/UFRGS, Porto Alegre, 231–274 (2000).
5. Paim, P. S. G., Wildner, W., Chemale Jr, F. Estágios evolutivos da Bacia do Camaquã (RS). *Ciência e Natura* 1-36. (2014).
6. Borba, A. W., Mizusaki, A. M. P., Maraschin, A. J., Silva, D. R. A. Santa Barbara Formation (Early Paleozoic, Cacapava do Sul, southern Brazil): Petrographic and Sm Nd isotopic provenance parameters. *Journal of South American Earth Sciences* 26, 485–497 (2008).
7. Borba, A. W., Mizusaki, A. M. P., Silva, D. R. A., Koester, E., Noronha, F. L., Casagrande, J. Provenance of the Neoproterozoic Maricá Formation (Sul-rio-grandense Shield, Southern Brazil): Petrographic and Sm-Nd isotopic constraints. *Gondwana Research* 9, 464–474 (2006).
8. Almeida, R. P., Santos, M. G. M., Fragoso-Cesar, A. R. S., Janikian, L., Fambrini, G. L. Recurring extensional and strike-slip tectonics after the Neoproterozoic collisional events in the southern Mantiqueira province. *An Acad. Bras Ciências* 84 (2), 347–376 (2012).
9. Wildner, W., Lima, E. F., Nardi, L. V. S., Sommer, C. A. Volcanic cycles and setting in the Neoproterozoic III to Ordovician Camaquã Basin succession in southern Brazil: characteristics of post-collisional magmatism. *J. Volcanol. Geotherm. Res*. 118, 261-283 (2002).
10. Janikian, L., Almeida, *et al.* The continental record of Ediacaran volcano-sedimentary successions in southern Brazil and their global implications. *Terra Nova* 20, 259–266 (2008).
11. Lehn, I., Fallgatter, C., Kern, H. P., Paim, P. S. G. Co-genetic, cohesive and non-cohesive delta front facies: A case study of flow transformation in a lacustrine setting, Camaquã Basin, southernmost Brazil. *Journal of South American Earth Sciences* 86, 271–286. (2018).
12. Fambrini, G. L., Janikian, L., Almeida, R. P., Fragoso-Cesar, A. R. S. O Grupo Santa Bárbara (Ediacarano) na Sub-Bacia Camaquã Central, RS: estratigrafia e sistemas deposicionais. *Revista Brasileira de Geociências*, 35, 2, 227-238 (2005).
13. Marconato, A., Almeida, R. P., Turra, B. B., Fragoso-Cesar, A. R. S. Pre-vegetation fluvial floodplains and channel-belts in the Late Neoproterozoic-Cambrian Santa Bárbara Group (Southern Brazil). *Sedimentary Geology*, 300, 49-61 (2014).
14. Borba, A. W., Mizusaki, A. M. P. Santa Bárbara Formation (Caçapava do Sul, southern Brazil): depositional sequences and evolution of an Early Paleozoic postcolisional basin. *J. S. Am. Earth Sci*. 16 (5), 365–380 (2003).
